# Using deepfakes for experiments in the social sciences - A pilot study

**DOI:** 10.3389/fsoc.2022.907199

**Published:** 2022-11-29

**Authors:** Andreas Eberl, Juliane Kühn, Tobias Wolbring

**Affiliations:** Chair of Empirical Economic Sociology, Friedrich-Alexander University Erlangen-Nürnberg, Nuremberg, Germany

**Keywords:** deepfakes, face swap, deep learning, experiment, physical attractiveness, student evaluations of teachers

## Abstract

The advent of deepfakes - the manipulation of audio records, images and videos based on deep learning techniques - has important implications for science and society. Current studies focus primarily on the detection and dangers of deepfakes. In contrast, less attention is paid to the potential of this technology for substantive research - particularly as an approach for controlled experimental manipulations in the social sciences. In this paper, we aim to fill this research gap and argue that deepfakes can be a valuable tool for conducting social science experiments. To demonstrate some of the potentials and pitfalls of deepfakes, we conducted a pilot study on the effects of physical attractiveness on student evaluations of teachers. To this end, we created a deepfake video varying the physical attractiveness of the instructor as compared to the original video and asked students to rate the presentation and instructor. First, our results show that social scientists without special knowledge in computer science can successfully create a credible deepfake within reasonable time. Student ratings of the quality of the two videos were comparable and students did not detect the deepfake. Second, we use deepfakes to examine a substantive research question: whether there are differences in the ratings of a physically more and a physically less attractive instructor. Our suggestive evidence points toward a beauty penalty. Thus, our study supports the idea that deepfakes can be used to introduce systematic variations into experiments while offering a high degree of experimental control. Finally, we discuss the feasibility of deepfakes as an experimental manipulation and the ethical challenges of using deepfakes in experiments.

## Introduction

Since the end of 2017, the creation and distribution of deepfakes have increased sharply. This phenomenon started on the platform *reddit* with a user called “deepfake” - a symbiosis between *deep learning* and *fakes*- who created the same name forum on this platform. By making the computer code available, other users could produce deepfakes themselves and contribute their results through the platform, leading to their immense popularity (Kietzmann et al., 2020). Besides the manipulation of audio records, deepfakes provide the ability to swap one person's face onto another in a picture or a video based on artificial intelligence applying deep learning techniques. The specific algorithm, which creates these fake videos, learns and improves by constantly mimicking gestures and facial expressions (Maras and Alexandrou, 2019). While image editing packages have only enabled adding, replicating, or removing objects on images (Verdoliva, 2020), *video manipulations* become more realistic using artificial intelligence. The most common examples are videos that include celebrities or politicians whose faces have been swapped with those of other persons or individuals whose facial attributes or styles (e.g., hair) have been altered (Langguth et al., 2021). Moreover, deepfakes also include sophisticated *image manipulations* based on artificial intelligence. Besides the possibility to create images based on the semantic layout (Park et al., 2019), sketches (Isola et al., 2017), or text (Reed et al., 2016), it is also feasible to modify images, such as changing the color scheme (Zhu et al., 2017) or background (Isola et al., 2017), without affecting their realistic perception (see also Tolosana et al., 2020).

This high degree of realism of deepfakes and their indistinguishability from original videos and images for the inattentive human mind lead to the perception of deepfakes as a threat to human society, democracy, and public discourse as well as a potential driver of societal radicalization, polarization and conflict (Borges et al., 2019; Qayyum et al., 2019; Westerlund, 2019). Therefore, it is not surprising that buzzwords like manipulation, abuse, and political influence often appear in news reports and scientific pieces covering deepfakes. Examples could especially be seen in the U.S., where deepfakes were used to spread fake news (Westerlund, 2019). Having those examples from everyday life in mind, many argue that threats related to deepfakes outweigh their benefits (e.g., Fallis, 2020). While deepfakes carry the potential of disinformation and manipulative use, they cannot be dismissed exclusively as a threat, because differentiations exist concerning their ethical principles. As de Ruiter (2021) puts it: “deepfake technology and deepfakes are morally suspect, but not inherently morally wrong” (p. 1328). In her opinion, three factors condition the immoral use of deepfakes: representation of persons to which they would not consent, deliberate deception of viewers, and harmful intention. Considering these specific factors, a morally acceptable use of deepfakes is not entirely out of the question. Nevertheless, are deepfakes solely a threat to social cohesion or can they also help to advance social science knowledge?

Until now, most scientific papers dealing with deepfakes focus either on the extension of algorithms to improve the graphical results, solutions to detect those deepfakes, or their threats to society. However, less attention is paid to the potential of deepfakes for substantive research - especially as an approach for experimental manipulation with a high degree of control in the social sciences. In this paper, we aim to address this research gap and argue that deepfakes can be a valuable tool for conducting social science experiments. To demonstrate some of the potentials and pitfalls of deepfakes, we conducted a pilot study on the effects of physical attractiveness on students' evaluations of teaching. For this purpose, a deepfake video was created from two individuals with varying physical attractiveness. Students watched one of the two randomly assigned videos and rated the presentation, the instructor, and the video. Besides providing suggestive evidence on potential mechanisms of discrimination at work, we also conducted this experiment as an attempt to test the possibility of using the deepfake technology for experimental variation in sociological research. However, before we go into the details of this pilot study, we discuss previous research that has used deepfakes for answering social science research questions. While - to the best of our knowledge - only one study exists which uses deepfake videos in a similar way as our pilot (Haut et al., 2021), providing some background on existing research hopefully contributes to a better understanding of the potentials and pitfalls of the technique in the social sciences.

## Previous studies using deepfakes

The amount of literature on deepfakes has increased sharply since 2017, and many of these papers warn primarily about their dangers (e.g., Fallis, 2020). Rather than just reporting on these threats to society and democracy, we will take a broader social science perspective in this paper. Therefore, we will also address the potential of deepfakes in scientific research. Accordingly, this section also covers studies that used deepfakes as a treatment in experiments or surveys to answer social science research questions. Please note that this is not a systematic review (for systematic reviews on deepfakes, see: Westerlund, 2019; Gamage et al., 2021; Godulla et al., 2021).

Due to the threat potential attributed to deepfakes, several studies deal with the computer-assisted *detection of deepfakes*, i.e., automated detection through machine learning (e.g., Zhang et al., 2017; Matern et al., 2019; Fagni et al., 2021; Mehta et al., 2021; Trinh and Liu, 2021). Other studies focus on human detection of deepfakes by conducting experiments to determine whether individuals can reliably detect deepfaked content (images and videos). The upshot of these studies is that individuals fail to detect deepfaked images. For example, Nightingale and Farid (2022) show that artificial intelligence (AI) synthesized faces are indistinguishable from real faces. Experiments using manipulated videos point in the same direction corroborating the claim that people cannot reliably detect deepfakes (Khodabakhsh et al., 2019; Köbis et al., 2021; Ternovski et al., 2021). Possible reasons for this insufficient detection rate are that deepfakes are sometimes perceived as more authentic than the original videos (Köbis et al., 2021) and that AI-synthesized images are perceived as more trustworthy than real faces (Nightingale and Farid, 2022).

Deepfakes can therefore be seen as a further step as compared to manipulations that only have a *human-like* appearance, such as robots or avatars. A distinction in this respect is made by de Ruiter (2021): “While real person deepfakes attribute digitally produced forms of speech and behavior to real individuals, avatar[s] […] attribute actual speech and behavior of real persons to digitally produced avatars” (p. 1316). Nevertheless, researchers claim that head-talking avatars also reduce confidence in AI-generated results, while uncanny valley expectations act as a mediator (Weisman and Peña, 2021). The term “uncanny valley” refers to the feeling of unease due to conflicting information resulting from visual impressions that are neither clearly artificial nor clearly human (Mori et al., 2012). In this ambiguous context, two options arise, either the avoidance of human likeness (so that robots are clearly recognized as such) or the perfectionism of human likeness (so that robots cannot be distinguished from humans) (Welker et al., 2020). For the latter, deepfakes seem to be a suitable means.

However, deepfakes not only help to overcome eerie feelings, but they also show *influence on (social) media and trust*. For example, Vaccari and Chadwick (2020) use an existing political deepfake video (Obama/Peele video) in their experiment to investigate whether deepfakes are recognized as such by individuals and how this affects respondents' trust in the media. The results show that political deepfakes do not deceive individuals because they realize that the person in the video would never have said anything like that. However, watching the deepfake video increases uncertainty, reducing general trust in social media and the news. This finding is supported by Ahmed (2021), who uses survey data and shows that skepticism toward the media is increasing due to deepfakes. Going one step further, Dobber et al. (2021) investigate in an online experiment how a political deepfake (manipulated video and audio) affects political attitudes. The results indicate that deepfakes could be used to stage a political scandal. While attitudes toward the depicted politician are significantly lower after watching the deepfake video, attitudes toward the politician's party are not affected. Additionally, the authors show that political microtargeting techniques can intensify the effects of a deepfake. More general, the results by Hughes et al. (2021) suggest that deepfake videos influence viewers' attitudes and intentions in the same way, as is true for original (not faked) videos.

A simple solution to buffer the harmful consequences of deepfakes could be to raise awareness for the existence of deepfakes. However, warning individuals of deepfakes can further decrease trust in information and the media in general. In this context, Ternovski et al. (2021) use online experiments to warn voters of the existence and dangers of deepfakes before watching selected political videos. After receiving a warning regarding deepfake videos, the results show that individuals begin to distrust all political video footage presented in the experiment, even the original (not faked) videos. Thus, their results illustrate that deepfakes pose a problem not simply through the spread of misinformation but also through the delegitimization of true information.

To the best of our knowledge, there is only one study that leverages deepfakes for examining discrimination. Haut et al. (2021) show an image of a black person vs. an image of a white person using the same audio record in their experiment. The authors measure credibility as the percent of participants who believed the speaker was telling the truth. The results reveal that changing a person's race in a static image has no impact on credibility. In a second step, Haut et al. (2021) test the effect of showing either an original video or a manipulated video where the person's appearance in the original video is manipulated to appear more “white.” The original video shows a South Asian speaker, whereas the altered video shows a more “white” speaker. Unlike the presentation of an image, manipulation in a video significantly increases credibility.

To sum up, this literature review reveals that only a limited number of studies used deepfakes to investigate social science research questions beyond their effects on trust in media and politics. While previous research has mainly focused on the dangers of deepfakes or their detection by algorithms or humans, few studies address their potential, e.g., to study the discrimination of different groups of people like Haut et al. (2021). However, this lack of studies is surprising, as deepfakes have specific advantages for social science research. Deepfakes enable the systematic variation of visual and audio stimulus materials in experiments, while holding all else constant. In particular, the simultaneous manipulation of visual and acoustic materials represents an extension of previous techniques. For example, researchers can manipulate a person's face while keeping all other video elements like the audio record and its speed, background, clothing, and hairstyles identical. Influences outside the individual, which also affect their perception (Keres and Chartier, 2016), can be kept stable across experimental conditions, minimizing biases in estimates of physical attractiveness effects. Consequently, deepfakes offer a high degree of experimental control and thus appear to be a promising method to identify causal effects in experiments by systematically varying only one factor at a time.

## Motivation and theoretical background of pilot study

In order to fill the research gap identified in the previous section, we conducted a pilot study to explore the feasibility of using deepfakes for social science research, especially experiments on discrimination. In this pilot, we build on previous research of one of the authors (Wolbring and Riordan, 2016) on the effects of instructors' physical attractiveness on students' evaluations of teaching (SET). The basic idea is that physical attractive instructors might profit from a *beauty premium* in the form of better SET scores (e.g., Hamermesh and Parker, 2005). Different theoretical mechanisms might cause this effect, including an attention boost to physical attractive instructors (e.g., Mulford et al., 1998), the ascription of positive stereotypes to good looking faculty (e.g., Dion et al., 1972) and the beauty glamor effect which can buffer the consequences of misconduct and bad performance to some degree (e.g., Bassili, 1981). However, this premium can also turn into a *beauty penalty* (e.g., Andreoni and Petrie, 2008) if positive stereotypes are disappointed by conflicting behavior or if the activated stereotypes do not match with the demands of the context (e.g., physically attractive female managers). Thereby, current research shows for various contexts that men consistently benefit from physical attractiveness, while the picture is more differentiated for women, who may profit or be disadvantaged (Hosoda et al., 2003; Paustian-Underdahl and Walker, 2016; Pajunen et al., 2021).

In the current literature on the effect of physical attractiveness on teaching evaluations, there are two opposing approaches. To some extent, our approach takes a middle ground combining strengths from both approaches. One group of studies relies on field data collected in real teaching contexts (Felton et al., 2008). The other group of studies uses experimental data collected in the context of laboratory experiments (Wolbring and Riordan, 2016). While in the first approach, based on observational data, the singular effect of physical attractiveness is hard to separate from other nuisances, in the second approach, based on experimental data, there is no real classroom situation. So far, image and audio material had to be separated from each other, as their simultaneous manipulation was not possible.

This is where the deepfake technology comes in, bringing exactly this advantage. By using deepfakes, it is possible both to vary the physical attractiveness in a targeted manner and to combine manipulated image material with an audio record, thus creating a realistic (online) teaching situation. Thereby, deepfakes can also account for the fact that some researchers assume that the evaluation of static and dynamic faces is based on different evaluation schemes (Riggio et al., 1991; Rubenstein, 2005). In order to achieve the most realistic assessment of teaching, we argue that videos should be given preference over images. Another advantage is a high degree of experimental control which helps to isolate the effect of physical attractiveness, since the audio records and background conditions of the original and the deepfake are identical, while other nuisances are addressed by means of randomization.

Guided by our theoretical framework, we created a deepfake based on two persons with varying physical attractiveness and conducted a small experiment among student subjects. In the experiment, we focus, on the one hand, on practical and methodological aspects such as the effort needed to manipulate the videos for social scientists without a strong background in computer science, the challenges we encountered when implementing the deepfakes, and the realism of the resulting videos according to participants of the study. On the other hand, we provide suggestive evidence on a substantive research question by exploring whether there are differences in the SET scores of a more and of a less physically attractive instructor. Given that the deepfakes allow us to control all other nuisances, finding such differences would point toward a beauty premium or beauty penalty. However, it is important to note that this is only suggestive evidence due to the small number of videos (*N* = 2) and subjects (*N* = 37) which also limits the possibilities to dig deeper into the underlying mechanisms at work.

## Creation of the deepfakes

For the creation of the deepfake video, we used the software *deepfacelab*^1^ which relies on the principle of an autoencoder, a special type of neural network. Thereby, an image first passes through an *encoder* that compresses the information provided, resulting in a low-dimensional representation of that input. On this basis the *decoder* tries to restore the original image (Perov et al., 2020). Using this technology, we can systematically vary the stimulus material shown in our experiment. As a starting point, we used the video of a person who is perceived as physically more attractive (original A) and the video of a person who is perceived as physically less attractive (original B) as the source materials. In the creation of the deepfake, we insert the face of the latter (B) into the video of the physically more attractive person (A) resulting in a deepfake.

In order to check whether the instructors actually differ in terms of their physical attractiveness, the pictures of those two individuals were evaluated in advance. So on the one hand, person A of the original video and on the other hand, person B whose face will be used for the creation of the deepfake. In order to avoid suspicion among the participants of the actual experiment on the deepfaking of videos, we asked 32 external reviewers from a snowball sample in our personal network for physical attractiveness ratings on a seven-point Likert scale from 1 = not at all physically attractive to 7 = very physically attractive. Each reviewer only rated one picture to avoid mutual influence or anchor effects. The physical attractiveness ratings of person A and B differ by almost two scale points (mean for person A: 4.93, mean for person B: 3.06).

In a next step, we recorded the videos and generated the deepfakes. To facilitate the creation of the deepfake, both videos were shot under the same conditions (e.g., camera position, recording device, etc.) and with neutral background. After the recording, we switched to *deepfacelab* for extracting the images from both videos. We then started the training running three weeks^2^ until we reached over 250,000 iterations. For the merging, we adjusted the corresponding settings (size of the mask, face size, color, etc.) so that the deepfake becomes as realistic as possible. After the complete merge, we visually checked that no artifacts were visible in the deepfake video^3^. Following this procedure, we carried out various tests in order to be able to select the best result and gain experience with the software. The decisive factors for choosing the final video were, on the one hand, that the deepfake appears as credible and convincing as possible, and no visual artifacts are recognizable. On the other hand, a second important criterion was that the people in the source material are rated as differently as possible concerning their physical attractiveness to secure sufficient variation in physical attractiveness. So, if there are differences in the ratings of the videos, we can likely attribute them to the different appearances of the individuals.

**Figure 1:**
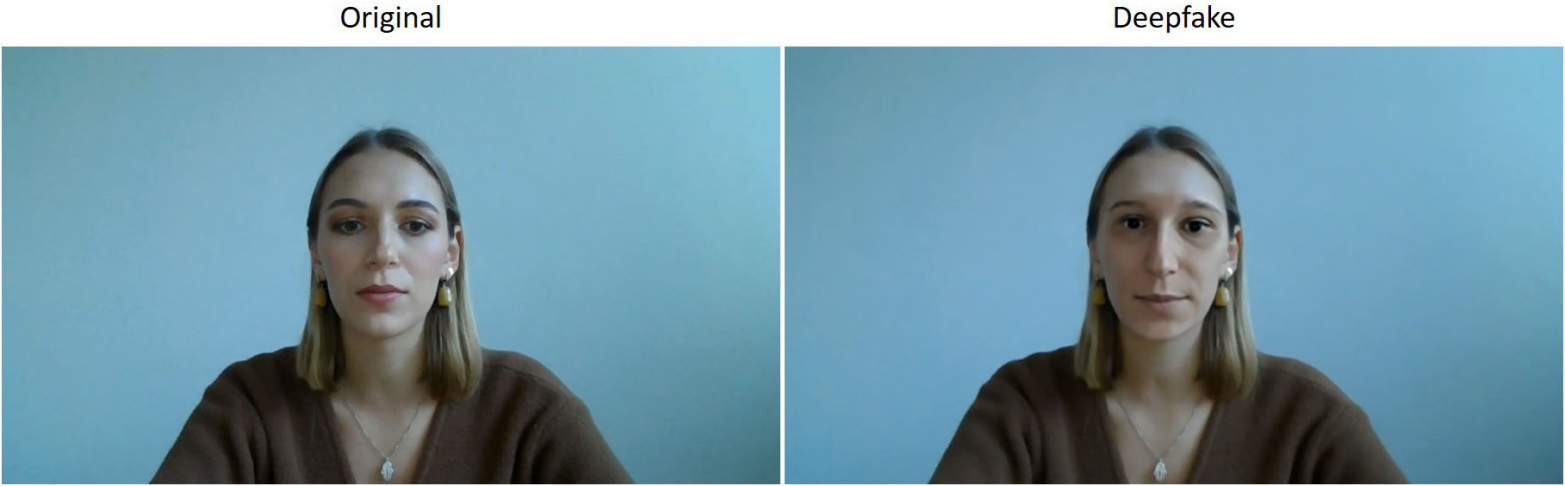
Experimental stimuli = **Original**: Physically more attractive instructor; **Deepfake**: physically less attractive instructor.

In order to ensure that the person depicted in the deepfake video was indeed physically less attractive than the person in the original video, we also asked 18 external reviewers to rate the physical attractiveness of the hypothetical person shown in the deepfake. This rating matches almost perfectly to the one of the physically less attractive person (mean for person in deepfake: 2.89 as compared to 3.06 for the real person B). With those results, we can ensure that the treatment group evaluates the physically less attractive person (deepfake), while the control group assesses the physically more attractive person (original). An image of the stimulus material used in the experiment is shown in Figure 1 (videos in German language are available upon request).

## Experimental setting and questionnaire

The experiment was embedded in an online bachelor course at Friedrich-Alexander University Erlangen-Nürnberg with 39 students. All students received the same instruction, explaining that after watching a video of a hypothetical teaching situation, they would have to rate both the presentation and the instructor. By having only one person giving the instruction, interviewer effects were avoided^4^. A voucher worth 20 euros was raffled among the students who performed best in a test on the content of the presentation. In this way, we wanted to ensure that the students focus on the content of the video.

After one instruction for all respondents, the students were randomly assigned into one of two groups (after data cleaning: original *N* = 19, deepfake *N* = 18) and were not able to switch between groups. The treatment group watched the deepfake video, which contained the physically less attractive instructor, and the control group watched the original video with the physically more attractive instructor. Accordingly, each participant watched either the deepfake video *or* the original video. The 2-minute video was an introduction to the topic of social inequality based on Solga et al. (2009). Following the study by Wolbring and Riordan (2016), the students then received the corresponding SET questionnaire, which they filled out online and anonymously. At the end of the survey, respondents were redirected to another page for the lottery to ensure that personal information and survey responses cannot be linked.

The questionnaire started with the *evaluation of the presentation*, including four items - the structure of the presentation, its argumentation chain, its speed, as well as its effect on students' interest in the topic - in random order. Afterwards, the *evaluation of the instructor* was based on nine items, which were also displayed in random order. The instructor's competence was evaluated with two items, followed by questions on her rhetoric and leadership qualities. In addition, the students were asked to assess the instructor's preparation, reliability, likeability, open-mindedness, and enthusiasm for the subject. The ratings of the presentation and the instructor are based on a Likert scale from 1 = does not apply at all to 7 = fully applies. To complete this evaluation, students assigned an *overall grade* for both the presentation and the instructor with all values including decimals between 1.00 = poor to 5.00 = excellent. Finally, five *knowledge questions* as well as *questions about the experiment* and *socio-demographics* formed the last part of the survey.

After data quality control and the subsequent deletion of two persons from the sample (final *N* = 37), our analyses concerning covariate balance suggest that the *randomization* was successful. Regarding interest in the topic, differences in average ratings are less than half a scale point (on a seven-point Likert scale: *t* = 0.99; *p* = 0.34). Similarly, prior knowledge of the topic differs between the treatment and the control group by only half a scale point (*t* = 1.59; *p* = 0.12). Given those small differences, we checked the robustness of the reported results by controlling for interest, prior knowledge, and the number of correct test answers in a linear regression model. Despite the small number of cases, we follow the request of a reviewer to report results from significance testing, but want to emphasize that due to the low statistical power of our study the results of significance testing should be treated with caution. In particular, conclusions about statistical significance should not be mixed with the strength of substantial relevance of an effect (Bernardi et al., 2017).

## Results on the credibility of the deepfake video

In order to evaluate whether we were able to generate a credible deepfake for the experiment, the subsequent analyses in this section focus on three aspects. First, we asked the respondents to summarize the *study's aim* in their own words. On the one hand, part of the answers by the students referred to the content of the presentation, namely “social inequality.” On the other hand, part of the students suspected that this study was about teaching evaluations. None of the answers addressed the video itself, nor did any comment suspect a possible manipulation of the instructor.

**Figure 2:**
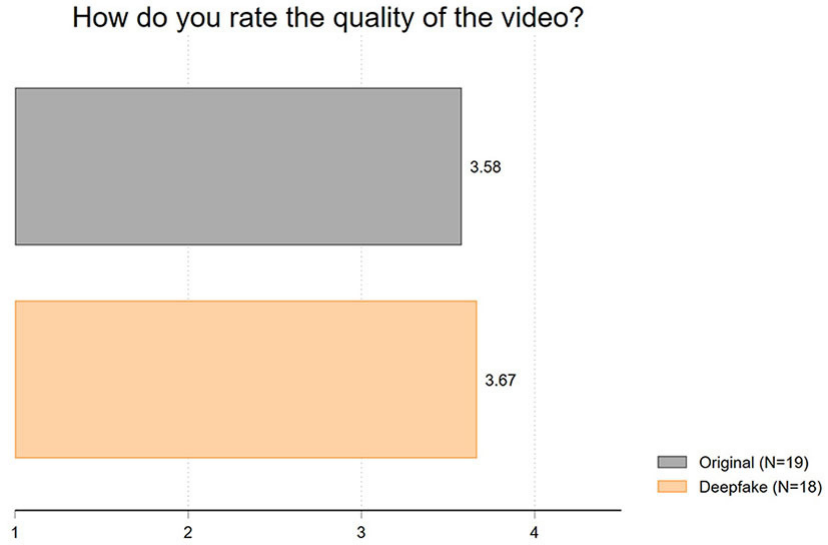
Mean values of video quality.

Second, we evaluated the *video quality*. In this context, we suspected that the deepfake may not be obvious to the students but that they may notice a deteriorating quality, for example, by perceiving the video as jerky or distorted. However, the results displayed in Figure 2 show that the video quality is rated comparably in both conditions. The average ratings of the video quality (1 = very poor to 7 = very good) hardly differ, with a difference of 0.1 (mean of original: 3.58; mean of deepfake: 3.67; *t* = 0.23; *p* = 0.82).

**Figure 3:**
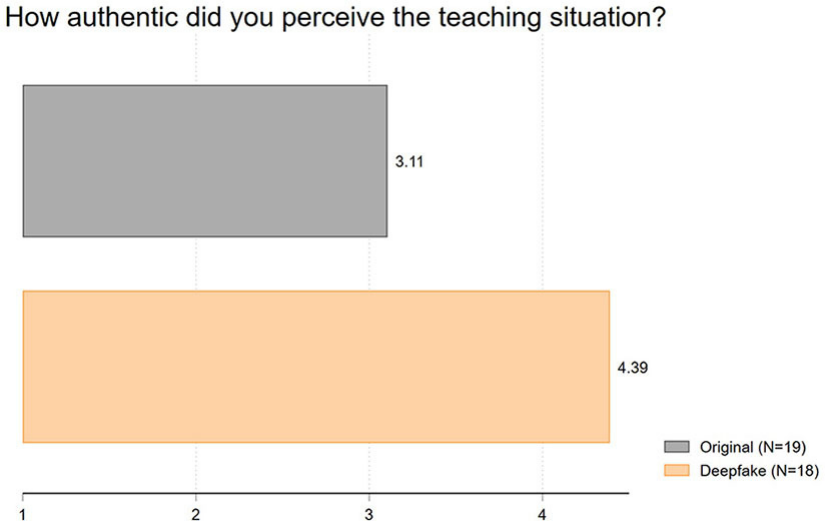
Mean values of authenticity.

Finally, we analyzed the perceived *authenticity* following Haut et al. (2021). This question generated the largest differences among all inspected variables, although surprisingly not in the expected way: the deepfake video was rated more authentic than the original video differing by 1.28 scale points (1 = not authentic at all to 7 = very authentic). Accordingly, we find the same effect with regard to authenticity as Köbis et al. (2021) in their study. As Figure 3 shows, the average authenticity of the original video was rated at 3.11, while the deepfake video was rated with a mean value of 4.39 (*t* = 2.93; *p* = 0.01).^5^

To sum up, we find no indications that the deepfake was detected by the participating students or that the deepfake video was perceived of lower quality than the original video. The deepfake also was not perceived as less authentic, although - as explained in footnote 5 - some concerns remain regarding the exact meaning of this authenticity measure in our study.

## Suggestive evidence on the effects of attractiveness on SET

**Figure 4:**
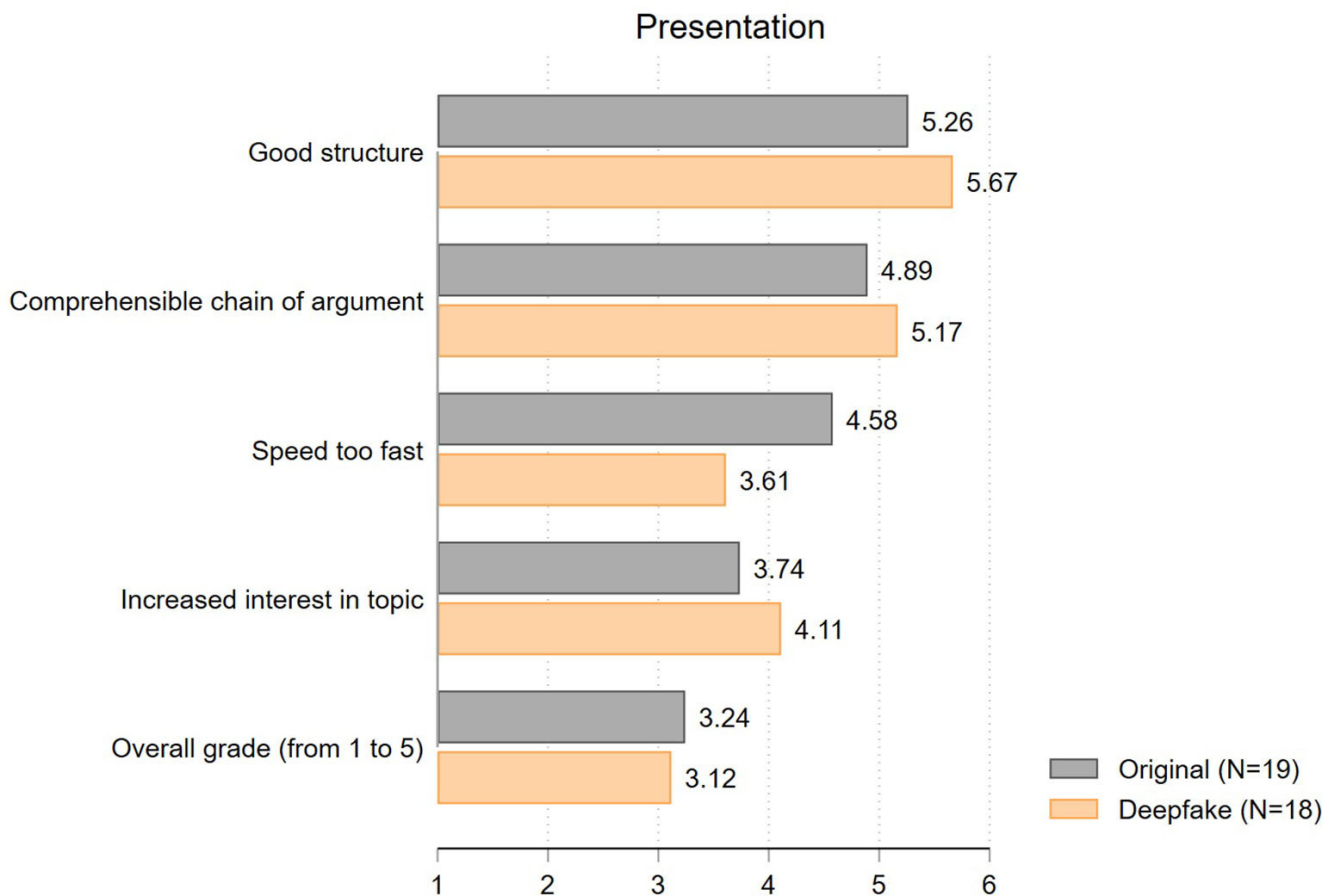
Evaluation of presentation (mean values).

Having ruled out concerns about the potential detection of deepfakes, we can now explore whether differences in the evaluations between treatment and control group using deepfakes exist and whether they point toward a beauty premium or penalty. First, we present the results of students' *evaluation of the presentation*. Since we used the identical video in both experimental groups except for the persons' face, we expect the presentation ratings to be very similar. Our results largely confirm this expectation. As displayed in Figure 4, almost all presentation ratings of the deepfake and the original video differ by no more than half a scale point (*structure*: *t* = 0.83; *p* = 0.41; *comprehensible argumentation*: *t* = 0.59; *p* = 0.56; *impact on interest*: *t* = 0.76; *p* = 0.45). In line with this, the overall ratings of the presentation in grades (from 1.00 = poor to 5.00 = excellent) only slightly differ between the two experimental groups (3.24 vs. 3.12; *t* = 0.49; *p* = 0.63). The only exception concerns student's rating of the speed of the presentation. Surprisingly, students especially expressed that the speed of the original video is too fast compared to the deepfake (mean: 4.58 vs. 3.61; *t* = 1.53; *p* = 0.13). Even if this difference is not statistically significant, the difference is remarkably large given that the original and the deepfake video are based on exactly the same source, involving the identical audio record. We interpret this result as a first indication that the beauty premium does not show in our experiment, while there might be a beauty penalty at work.

**Figure 5:**
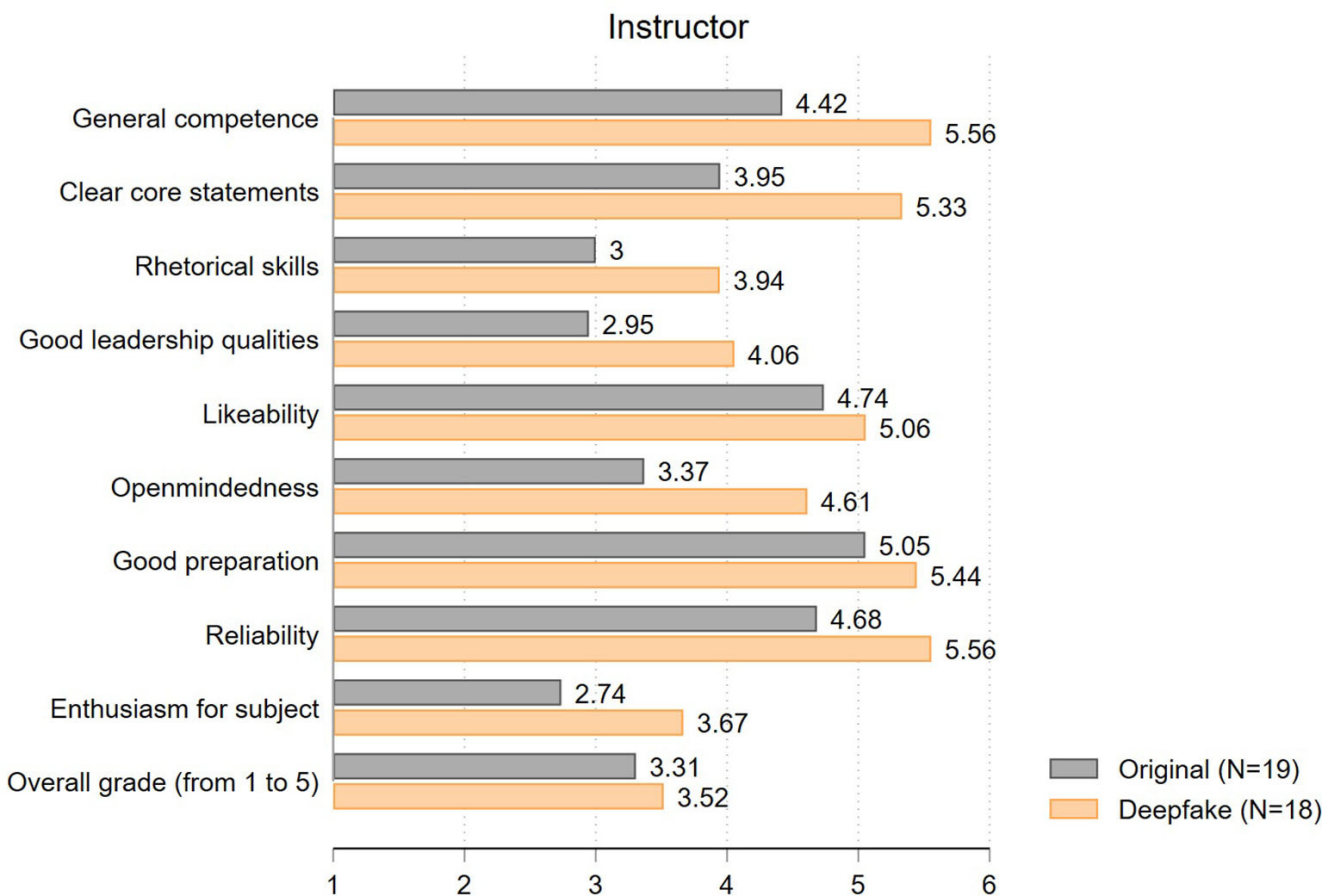
Evaluation of instructor (mean values).

Next, we focus on students' *evaluation of the instructor*, where we expect larger SET differences due to the face swap. The results are well in line with this suspicion (see Figure 5). Only the ratings for *likeability* (mean of original: 4.74, mean of deepfake: 5.06; *t* = 0.66; *p* = 0.52) and *good preparation* (5.05 vs. 5.44; *t* = 0.95; *p* = 0.35) are similar in the two groups. All other items differ by almost one scale point or more. The largest differences exist in ratings on *general competence* (4.42 vs. 5.56; *t* = 2.75; *p* = 0.01) and *clear core statements* (3.95 vs. 5.33; *t* = 2.41, *p* = 0.02). Likewise, the perception of the *rhetorical skills* (3.00 vs. 3.94; *t* = 1.66; *p* = 0.11), *leadership qualities* (2.95 vs. 4.06; *t* = 2.62; *p* = 0.01), *open-mindedness* (3.37 vs. 4.61; *t* = 2.14; *p* = 0.04) and *reliability* (4.68 vs. 5.56; *t* = 2.20; *p* = 0.03) are influenced by the appearance of the instructor. Overall, both instructors are perceived to show only average *enthusiasm for their subject*, although here, again, the degree of enthusiasm of the deepfake instructor is rated 0.93 scale points better (2.74 vs. 3.67; *t* = 1.69; *p* = 0.10). Taken together, we see more positive instructor ratings for the deepfake than for the original video. The analysis of the overall instructor ratings in grades (from 1.00 = poor to 5.00 = excellent) points in the same direction (3.31 vs. 3.52) - even though the overall grade does not differ significantly (*t* = 0.81; *p* = 0.42). Therefore, there is no evidence for the existence of a beauty premium here either, but rather some suggestive evidence for a beauty penalty.

## Discussion and implications

The nascent technology of deepfakes has important implications for the social sciences, both concerning its substantive research question such as misinformation and media trust and, as we contend, as a potential method for experimental manipulation. Our literature review shows that only a few social science studies address deepfakes while moving beyond the detection and dangers of this technology. In particular, deepfakes so far have been very rarely used as a tool for developing manipulations in experiments. We fill this research gap with our pilot study, and our findings suggest the feasibility of such an approach. Social scientists can successfully create a credible deepfake even without a corresponding education in computer science. Based on different test trials, we acquired appropriate knowledge in a reasonable amount of time that allowed us to create a deepfake using standard software and hardware. Notably, the quality of the deepfake was - in the eyes of the experimental subjects - comparable to our original video. None of the student subjects realized that they are watching a manipulated video.

Our study further underlines that deepfakes are suitable for researching social science issues in general and discrimination in particular. Because deepfakes maximize the videos' comparability (especially by having an identical audio record and the same conditions such as hairstyles, background, clothing, etc.), differences in ratings can be causally attributed to the varied stimulus. Using the case of physical attractiveness, our study supports the idea that deepfakes can be used to introduce systematic variations into experiments, while offering a high degree of experimental control. As a result, there are only small differences in students' evaluation of the presentation in the two videos, but larger differences in the evaluations of the two instructors. By holding all other factors constant, we can attribute these differences to the appearance of the instructors. However, in contrast to previous studies (e.g., Wolbring and Riordan, 2016), our suggestive evidence points to adverse effects of physical attractiveness. The physically less attractive instructor is rated better than the physically more attractive instructor. One possible way to reconcile this finding with previous results is that the physically more attractive instructor in the original video was rather young. Students might thus have perceived this physically attractive and young instructor as less authentic and competent than the physically less attractive but older instructor (see text footnote 5). However, as this study is based on a small sample, one should not overinterpret the fact that this suggestive evidence from the pilot study pointing toward a beauty penalty conflicts with existing large-scale studies documenting a beauty premium. A replication of our pilot with more subjects and videos is needed. While the homogeneity of our student sample is an advantage for a first test with a small sample, sampling from a broader student population with more diverse backgrounds and majors is worth considering. Such a more heterogeneous and representative sample would help to address concerns about sample selection and to answer questions about the generalizability of our results.

Besides these methodological and substantive insights from this pilot study, the use of deepfakes in social science studies raises more general practical and ethical challenges, concerns, and tensions associated with the application of this technology. Subjects are - by definition - deceived when deepfakes are used in studies *without* actively communicating their use. In the social and behavioral sciences, there are conflicting views on the appropriateness of deception for research purposes, ranging from complete rejection of deception on one side to reinforcement of the benefits associated with deception on the other side (Barrera and Simpson, 2012). Thus, deepfake technology appears to be morally problematic at first glance because it violates social norms like truthfulness and risks undermining people's autonomy.

However, although deepfakes may appear morally suspect, the technology is not inherently morally wrong and, as we contend, there are ways to use deepfakes in empirical research in responsible ways. According to de Ruiter (2021), three factors are important to determine whether deepfakes are morally problematic: (i) would the faked person complain about how she/he is portrayed; (ii) does the deepfake deceive the viewers; (iii) what is the intention with which the deepfake was created. In our study, the faked person was aware of the purpose of the study when videotaping the presentation. The intention of the deepfake was to investigate discrimination and did not cause any harm whatsoever. Finally, one might argue that the viewers were deceived in our study, but we explicitly informed our subjects that they are watching the video of a *hypothetical* teaching situation. Moreover, we decided not to inform subjects after the experiment because, as our literature review has shown, such an active communication that deepfakes are used can harm people's general trust in the media and politics.

Additionally, it can be argued that deepfakes are real enough to avoid a sense of eeriness, which other studies with robots or avatars have shown (de Borst and de Gelder, 2015; Konijn and Hoorn, 2020). On the one hand, the deepfake technology offers great advantages concerning the authenticity of used video materials, whereby this is accompanied by a pleasant feeling when viewing them - in comparison to the problematic feeling of eeriness watching a human-like robot or avatar. On the other hand, the use of this technology evokes the often-discussed danger that the difference between the original and the deepfake is no longer perceptible. In this context, a clear distinction is needed: (a) when are deepfakes used to manipulate and deceive people in order to create harm, so that the lack of distinguishability is also morally reprehensible. And (b) when are deepfakes used as a scientific instrument in order to create optimal experimental conditions. In the latter case, there is an opportunity to make the most of this development. The accompanying lack of distinctiveness creates the conditions for investigating the different treatment of *real* persons based on their appearance and, if applicable, the underlying discrimination mechanism.

While we believe that this approach has circumvented the major concerns when using deepfakes, other studies might warrant other avenues to address these issues. Therefore, more research is not only needed to further explore the possibilities of deepfakes for answering substantive research questions in the social sciences, but also to address the associated ethical challenges of using deepfakes in scientific experiments.

## Data availability statement

The data and code for this study are publicly and permanently available at the GESIS Datorium (Eberl et al., 2022).

## Ethics statement

Ethical review and approval was not required for the study on human participants in accordance with the local legislation and institutional requirements. Written informed consent for participation was not required for this study in accordance with the national legislation and the institutional requirements. Written informed consent was obtained from the individual(s) for the publication of any potentially identifiable images or data included in this article.

## Author contributions

All authors listed have made a substantial, direct, and intellectual contribution to the work and approved it for publication.

## Conflict of interest

The authors declare that the research was conducted in the absence of any commercial or financial relationships that could be construed as a potential conflict of interest.

## Publisher's note

All claims expressed in this article are solely those of the authors and do not necessarily represent those of their affiliated organizations, or those of the publisher, the editors and the reviewers. Any product that may be evaluated in this article, or claim that may be made by its manufacturer, is not guaranteed or endorsed by the publisher.

## Footnotes

^1^Here, we used *DeepFaceLab_DirectX12_build_11_20_2021.exe* which can be downloaded on https://github.com/iperov/DeepFaceLab/. Thereby we used the following procedure: 1) clear workspace; 2) extract images from video data_src; 3) extract images from video data_dst; 4) data_src faceset extract; 4.1) data_src view aligned result; 5) data_dst faceset extract; 5.1) data_dst view aligned results; 6) train Quick96; 7) merge Quick96. Advanced settings regarding the merging were determined via various test trials with different videos and people.

^2^Please note that this time varies with the hardware used and the exact specification of the algorithm.

^3^Visual artifacts are errors in deepfakes, such as brief moments in which the original face is recognizable or visible attributes in the deepfaked face that belong to the original face, such as eyebrows or earrings (Verdoliva, 2020). In this context, attentive viewing of the videos by several people has proven to be a suitable method for us.

^4^In order to be able to respond to any questions or problems the students might encounter, there was an experimenter present in each group. These persons had the same name in both conditions and had a switched-off camera to avoid different visual stimuli. The option to ask questions was not used in either subgroup. Influences of the experimenter can therefore be excluded.

^5^Compared to Haut et al. (2021), we asked about the authenticity of the situation presented rather than the authenticity of the video. By adapting this question to our experiment in order to prevent suspicion about a potential video manipulation, we shifted the focus from the video to the situation and might have changed the meaning of the underlying measure. The observed lack of authenticity of the original video could be due to the fact that the person shown was perceived as too young to be a fully trained instructor, while the person in the deepfake looked older and might fit better in student's mental script of university instructors.
